# Robust Path Planning via Deep Reinforcement Learning

**DOI:** 10.3390/s26092658

**Published:** 2026-04-24

**Authors:** Daeyeol Kang, Jongyoon Park, Pileun Kim

**Affiliations:** Artificial Intelligence, Korea Aerospace University, Goyang-si 10540, Republic of Korea

**Keywords:** reinforcement learning, robot navigation, path planning, TD3, APF

## Abstract

Deep reinforcement learning (DRL) for autonomous mobile robot navigation faces several inherent limitations. The stochastic nature of actions generated by DRL policies can undermine performance consistency, while inefficient exploration frequently delays the learning process or prevents the discovery of optimal solutions. This research aims to enhance the robustness of path planning by addressing these challenges. To achieve this goal, we propose a hybrid approach that integrates the flexible decision-making capabilities of deep reinforcement learning with the stability of traditional path planning. The proposed model adopts the Twin Delayed Deep Deterministic Policy Gradient (TD3) network as its base. Notably, we pre-process LiDAR point cloud data to extract only essential features for the state representation, thereby preventing performance degradation from high-dimensional inputs and improving computational efficiency. Our model optimizes the learning process through two core strategies. First, it prioritizes experience data generated during training based on negative rewards, guiding the model to learn more frequently from critical failures rather than redundant successes. Second, it dynamically compares the action proposed by the TD3 network with a goal-oriented action from a classical path-planning algorithm in real time. By selecting the action with the higher estimated value, the model guides the policy toward a stable and effective trajectory from the earliest stages of training. To validate the efficacy of our approach, we conducted simulation-based experiments comparing the performance of the proposed model with existing reinforcement learning networks. To ensure statistical significance and mitigate the impact of random initialization, all reported results are averaged over 10 independent runs with different random seeds. The results quantitatively demonstrate that our model achieves significantly higher and more stable reward values, confirming a robust improvement in the path-planning process.

## 1. Introduction

The field of robotics has long been recognized for its contributions to providing practical convenience in human life, and has garnered significant attention worldwide. Early robots were primarily designed for stationary, repetitive tasks within structured industrial environments. However, recent research has increasingly focused on mobile robots capable of performing autonomous and diverse missions in dynamic and unstructured settings, built upon platforms such as autonomous vehicles, unmanned aerial vehicles (drones), and service robots.

With the advancement of Artificial Intelligence (AI), various data-driven models have emerged to tackle the complexities of autonomous navigation. While supervised and unsupervised learning approaches exhibit performance variations that heavily depend on the volume and quality of labeled data, reinforcement learning (RL) has gained prominence as a paradigm that allows an agent to learn optimal behaviors through direct interaction with its environment. This capability to leverage sensors and simulations for trial-and-error learning makes RL a focal point in current robotics research [1,2].

Traditional path-planning technologies, such as the A* algorithm and Dijkstra’s algorithm, enable navigation by deducing optimal routes from maps to generate the shortest, most efficient paths. However, these methods rely on complete and accurate environmental maps, lack robustness in dynamic situations with unforeseen obstacles, and are often limited to simplistic decision-making that does not account for complex robot dynamics [3]. To overcome these limitations, techniques employing neural networks have recently gained traction. In particular, RL-based navigation is expected to have a profound impact on mobile robot automation, as it can execute the entire sequence from perception to decision-making and control through a single, end-to-end network. This framework allows various sensor inputs, such as LiDAR and camera data, to be processed as state variables, which subsequently inform perception and judgment to output control actions [4].

Despite its promise, RL-based control faces significant challenges, including slow convergence, sample inefficiency, and instability in real-world deployments. A primary drawback is that probabilistically selecting actions from a learned policy can lead to inefficient or suboptimal paths, particularly during the early stages of training. The random initialization of network weight parameters, while facilitating broad exploration and the collection of diverse samples for Q-value estimation, can also misguide the agent to learn suboptimal routes or fail to achieve convergence entirely [5,6].

Furthermore, reinforcement learning, which is fundamentally based on the Bellman equation for value estimation, suffers from the “curse of dimensionality.” In an n-dimensional state space, even a moderately complex 10-dimensional space where each dimension is discretized into just 10 steps results in $10^{10}$ possible state combinations. It is computationally intractable for an agent to experience every state-action pair to learn an optimal policy [7]. Consequently, reducing the dimensionality of the state space is crucial for improving RL performance. This necessitates the development of methods to efficiently extract meaningful information from the high-dimensional data streams produced by sensors like LiDAR, translating them into compact and informative state representations.

To address these challenges, previous studies have actively explored hybrid architectures that combine the adaptability of RL with the stability of classical path planning. For instance, hierarchical approaches have been proposed where global planners, such as A* or Dijkstra, generate intermediate waypoints that guide the RL agent, effectively simplifying the navigation task into local goal tracking [8,9]. Other researchers have incorporated reactive methods, such as the Dynamic Window Approach (DWA) or Artificial Potential Fields (APF), to act as safety shields or to override RL actions in hazardous situations [10,11]. However, these existing methods often rely on rigid switching mechanisms between the planner and the network, which can lead to discontinuous control signals. Furthermore, an excessive reliance on classical planners may constrain the agent’s exploration, preventing it from learning flexible maneuvers necessary to escape complex local minima or dynamic obstacles that static planners fail to resolve. Recently comprehensive reviews [12] emphasize that addressing these rigid switching mechanisms through seamless hybrid integration, as well as enhancing real-time adaptability, is essential for next-generation autonomous navigation.

Unlike previous works that treat the classical and learning modules as separate or competing controllers, this study proposes a seamless integration of Deep Reinforcement Learning (DRL) with traditional path planning. We aim to overcome the performance inconsistencies arising from the probabilistic nature of actions generated by RL policies and the challenges of inefficient exploration. We achieve this by integrating DRL with traditional path planning, creating a hybrid system where the adaptive strengths of DRL and the stability of classical planners mutually compensate for their respective weaknesses. By combining the RL network with a path planner, we developed a framework that consistently learns a superior policy. To address the challenge of inconsistent performance due to inefficient exploration, our objective is to construct a reinforcement learning network that can be effectively applied to simulations and diverse environments by enabling more efficient exploration and strategic experience selection.

The primary contributions of this paper are threefold:

1. We propose a hybrid control architecture that integrates the Twin Delayed Deep Deterministic Policy Gradient (TD3) algorithm with the Artificial Potential Field (APF) path planner. The planner provides stable guidance by influencing the learning target, which accelerates convergence and improves policy robustness without stifling exploration.
2. We introduce a reward-based Prioritized Experience Replay (PER) mechanism that focuses training on experiences with high informational value (indicated by low rewards), efficiently addressing critical failure cases and substantially increasing learning efficiency.
3. We present a two-stage LiDAR point cloud preprocessing pipeline that effectively reduces the dimensionality of the sensory input into a compact, fixed-size state vector, mitigating the curse of dimensionality while preserving critical environmental information for safe navigation.

This paper is organized as follows. Section 2 reviews related work on the core technologies applied in this study. Section 3 provides a detailed description of the simulation environment and the proposed network architecture. Section 4 presents the experimental results, comparing our proposed method with baseline networks in a high-fidelity simulation. Finally, Section 5 concludes the paper with a summary of our findings and a discussion of future research directions.

## 2. Related Work

### 2.1. Artificial Potential Field (APF)

The Artificial Potential Field (APF) algorithm is one of the most widely used local path-planning methods for collision avoidance due to its simplicity, ease of implementation, and suitability for real-time robot operations. The APF method determines a path by conceptualizing the robot as a point particle moving within a force field. The path is generated by calculating the vector sum of two forces: an **attractive force** that pulls the robot toward a goal point and a **repulsive force** that pushes it away from obstacles.

Since this study investigates methods for efficiently reaching a goal while avoiding obstacles using deep reinforcement learning, the real-time, reactive nature of APF can be leveraged to complement our approach. Furthermore, APF introduces minimal computational overhead, thereby maintaining high network efficiency. Unlike global planners like the A* algorithm, APF can instantly generate a waypoint given only the current robot odometry, obstacle locations, and the goal position. This characteristic establishes a complementary relationship with reinforcement learning, which typically generates control actions at every time step.

The attractive force is derived from the Euclidean distance between the robot’s current position, *x*, and the goal position, $x_{g}$. The attractive potential function, $U_{\mathrm{att}}$, is typically defined as a quadratic function of this distance. A gain parameter, $k_{p}$, is introduced to adjust the weight of the function, as shown in Equation (1).

(1) $$U_{\mathrm{att}}\left(x,x_{g}\right)=\frac{1}{2}k_{p}{∥x-x_{g}∥}^{2}$$

To obtain the instantaneous attractive force, we take the negative gradient of the attractive potential energy. This results in a linear force vector pointing directly from the robot to the goal, as expressed in Equation (2).

(2) $$F_{\mathrm{att}}=-\nabla U_{\mathrm{att}}\left(x,x_{g}\right)=-k_{p}\left(x-x_{g}\right)$$

The repulsive force is a function that directs the robot away from obstacles by reflecting the distance to them. It is designed to react more strongly as the risk of collision increases, generating a larger force when the robot is closer to an obstacle and a smaller one when it is farther away. The repulsive potential energy, $U_{\mathrm{rep}}$, is proportional to the inverse difference between the distance to an obstacle, ρ, and a maximum influence distance, $\rho_{0}$. Similar to the attractive potential, a gain parameter, $k_{r}$, is used for adjustment. To reduce computational load and prevent interference with efficient pathfinding, obstacles beyond the distance $\rho_{0}$ are excluded from the calculation. This is formulated in Equation (3).

(3) $$U_{\mathrm{rep}}\left(\rho \right)=\left\{\begin{array}{cc} \frac{1}{2}k_{r}{\left(\frac{1}{\rho }-\frac{1}{\rho_{0}}\right)}^{2} & \mathrm{if}\;\rho \leq \rho_{0}\\ 0 & \mathrm{if}\;\rho >\rho_{0} \end{array}\right.$$

Similar to the attractive force, the repulsive force is obtained by taking the negative gradient of its potential energy, as shown in Equation (4).

(4) $$F_{\mathrm{rep}}\left(\rho \right)=-\nabla U_{\mathrm{rep}}\left(\rho \right)=\left\{\begin{array}{cc} k_{r}\left(\frac{1}{\rho }-\frac{1}{\rho_{0}}\right)\frac{1}{\rho^{2}}\nabla \rho & \mathrm{if}\;\rho \leq \rho_{0}\\ 0 & \mathrm{if}\;\rho >\rho_{0} \end{array}\right.$$

By summing the x and y components of the calculated attractive and total repulsive forces (summed over all nearby obstacles), a resultant force vector can be determined. This vector is then used to generate a waypoint for the robot’s navigation [13,14]. Despite its advantages, APF suffers from a major drawback: the robot can become trapped in local minima, such as within U-shaped obstacles or when attractive and repulsive forces perfectly cancel each other out. This limitation makes it an ideal candidate for integration with an explorative method like RL. Consequently, state-of-the-art hybrid frameworks literature actively leverage DRL to account for human-related uncertainties and dynamically adjust planning parameters, thereby circumventing local minima while retaining reactive safety [15].

### 2.2. Twin Delayed Deep Deterministic Policy Gradient (TD3)

**Figure 1:**
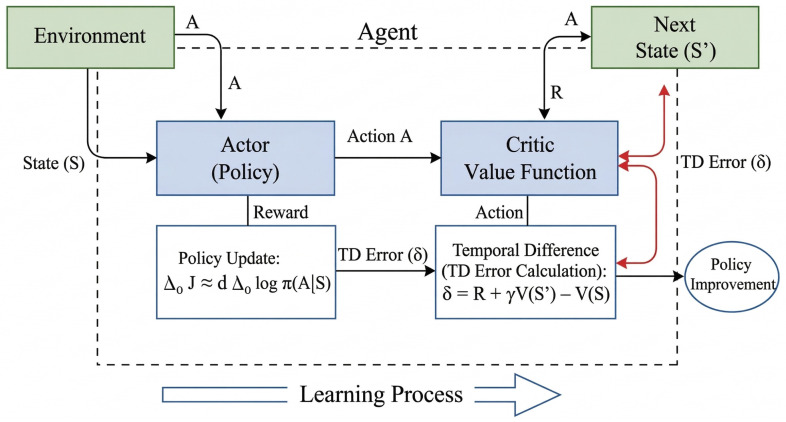
Actor-Critic Model.

The base DRL network utilized in this research is the Twin Delayed Deep Deterministic Policy Gradient (TD3) algorithm. TD3 is an off-policy, model-free algorithm designed for continuous action spaces and serves as a robust improvement over the Deep Deterministic Policy Gradient (DDPG) algorithm. It is fundamentally an Actor-Critic based model, where an ‘actor’ network learns the policy (mapping states to actions) and a ‘critic’ network learns the value function (the expected return of a state-action pair). The architecture of an Actor-Critic based RL framework is illustrated in Figure 1.

The TD3 network addresses several key instabilities present in DDPG by incorporating three distinctive features. First, it employs **Clipped Double Q-learning** to calculate the Temporal Difference (TD) error. Q-learning algorithms are known to suffer from overestimation bias, where estimated Q-values are systematically higher than true values. This occurs because the max operator used in the Bellman update can select overestimated action values, leading to a positive bias that propagates through the learning process. TD3 mitigates this by learning two independent critic networks and using the minimum of their Q-value predictions for the target update. The potential underestimation that can arise from this method does not significantly compromise overall performance.

Second, TD3 utilizes **Delayed Policy Updates**. In actor-critic methods, the policy (actor) and value (critic) networks are updated concurrently. If the critic network provides a noisy or inaccurate value estimate, the policy update can be misguided, leading to instability. With separate target policy and target critic networks, the TD error is reduced with each iteration of gradient descent, even when the TD target is fixed. TD3 operates on the principle that it is more efficient to update the policy after the critic network has sufficiently converged and the TD error is minimized. Therefore, the policy (and target networks) are updated less frequently than the critic networks (e.g., every two critic updates) to achieve better performance and stability.

Third, TD3 applies **Target Policy Smoothing Regularization**. Actor-critic methods can be sensitive to sharp peaks in the Q-function. To create a smoother value landscape, TD3 adds a small amount of clipped, sampled noise directly to the target action during the target Q-value calculation. The TD error is then calculated using the expected value over a mini-batch of these smoothed actions. This regularization technique ensures the learned policy is less likely to exploit errors in the Q-function and reduces the variance of the TD target, leading to more stable and efficient learning [16].

## 3. Methodology

### 3.1. Problem Formulation

We formulate the autonomous navigation task as a Markov Decision Process (MDP), defined by the tuple M=(S,A,P,R,γ). Here, S denotes the state space, A is the action space, P represents the state transition probability, R is the reward function, and γ is the discount factor. The objective of the reinforcement learning agent is to learn a policy $\pi_{\phi }\left(a_{t}|s_{t}\right)$ that maximizes the expected cumulative reward $E[\sum_{t=0}^{T}\gamma^{t}r_{t}]$.

#### 3.1.1. Robot Motion Model

The mobile robot in this study is modeled as a non-holonomic kinematic system operating on a 2D plane. We adopt the standard unicycle model to describe the robot’s motion. Let the robot’s pose at time step *t* be denoted by $p_{t}={\left[x_{t},y_{t},\theta_{t}\right]}^{T}$, where $\left(x_{t},y_{t}\right)$ represents Cartesian coordinates and $\theta_{t}$ is the heading orientation. The discrete-time kinematic equations are given by Equation (5):

(5) $$\begin{array}{c} x_{t+1}=x_{t}+v\cos\left(\theta_{t}\right)\Delta t\\ y_{t+1}=y_{t}+v\sin\left(\theta_{t}\right)\Delta t\\ \theta_{t+1}=\theta_{t}+\omega_{t}\Delta t \end{array}$$

In this framework, the linear velocity is determined adaptively based on the angular velocity $\omega_{t}$, and the agent controls only $\omega_{t}$. Therefore, the action space is one-dimensional.

#### 3.1.2. State Space

The state $s_{t}$ is designed to provide the agent with both local perception and global navigation information. The state consists of two components: the processed LiDAR voxel data and a vector of navigation states.

(6) $$s_{t}=\left(V_{t},x_{t}\right)$$

Here, $V_{t}\in R^{30\times 2}$ represents the voxel-based LiDAR features, where the raw point cloud is processed into 30 bins with 2 features each (specific details are presented in Section 3.4). The navigation state vector $x_{t}$ is defined as:

(7) $$x_{t}=\left[g_{x,t}^{local},g_{y,t}^{local},G_{x},G_{y},\theta_{t},\omega_{t-1}\right]$$

where $g_{x,t}^{local}$ and $g_{y,t}^{local}$ denote the goal coordinates transformed into the robot’s local coordinate frame, $G_{x}$ and $G_{y}$ are fixed global coordinates of the goal, $\theta_{t}$ is the robot’s current global yaw angle, and $\omega_{t-1}$ is the angular velocity from the previous time step.

#### 3.1.3. Action Space and Action Mapping

The action space A is continuous. While standard mobile robot control involves both linear velocity (*v*) and angular velocity (ω), we propose a low-dimensional action space mapping to ensure smooth navigation and narrow the search space for the RL agent. In this scheme, the network outputs a single scalar value corresponding to the angular velocity, while the linear velocity is determined adaptively based on the magnitude of the angular velocity.

**Figure 2:**
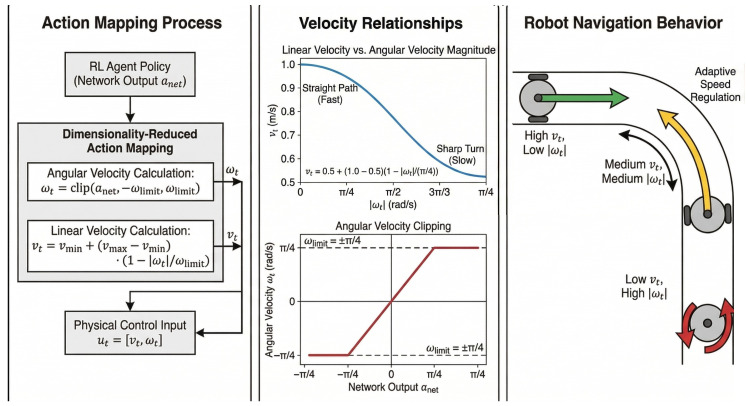
Action Mapping.

The angular velocity $\omega_{t}$ is clipped to the range $\left[-\frac{\pi }{4},\frac{\pi }{4}\right]$ rad/s. To ensure dynamic stability, the linear velocity $v_{t}$ is scaled inversely with $|\omega_{t}|$, constrained within $\left[v_{\min},v_{\max}\right]=\left[0.5,1.0\right]$ m/s. This mechanism ensures that the robot decelerates during sharp turns and accelerates on straight paths. The mapping functions are defined as:

(8) $$\omega_{t}=\mathrm{clip}(a_{\mathrm{net}},-\omega_{\mathrm{limit}},\omega_{\mathrm{limit}})$$

(9) $$v_{t}=v_{\min}+\left(v_{\max}-v_{\min}\right)\cdot \left(1-\frac{|\omega_{t}|}{\omega_{\mathrm{limit}}}\right)$$

where $\omega_{\mathrm{limit}}=\frac{\pi }{4}$. Consequently, although the physical control input is two-dimensional ($u_{t}=\left[v_{t},\omega_{t}\right]$), the effective action learned by the policy focuses on steering, with speed being automatically regulated to maintain dynamic stability. This action mapping scheme is illustrated in Figure 2.

#### 3.1.4. Reward Function

The reward function R is designed to guide the robot to the goal while avoiding collisions and optimizing path efficiency. It consists of a distance-based reward, a progress reward, and collision/time penalties. The detailed mathematical formulation of the proposed reward structure is presented in Section 3.2.

### 3.2. Proposed Reward Function

The design of the reward function is paramount to the success of a reinforcement learning agent. The TD-error is generated from the difference between the target Q-value and the current Q-value. A sparse reward function, as shown in Equation (10), could address this by assigning a large positive reward for reaching the goal, a large negative reward for a collision, and a small penalty for each step taken to encourage efficiency.

(10) $$R_{\mathrm{sparse}}=\left\{\begin{array}{cc} +100 & \mathrm{if}\;\mathrm{goal}\;\mathrm{is}\;\mathrm{reached}\\ -100 & \mathrm{if}\;a\;\mathrm{collision}\;\mathrm{occurs}\\ -5 & \mathrm{else} \end{array}\right.$$

However, sparse rewards provide infrequent feedback, which can make learning extremely difficult in complex environments. Because the TD-error is calculated at every time step, a dense, step-by-step reward signal significantly enhances the learning efficiency of the critic network by providing continuous feedback. This approach, known as reward shaping, requires careful design to prevent the agent from exploiting the reward function in unintended ways (reward hacking) while enabling a more granular representation of Q-values.

To this end, we designed a dense reward function based on the following principles:

1. **Proximity to Goal:** A higher reward is granted as the Euclidean distance between the robot’s current position and the goal decreases.
2. **Path Efficiency:** The reward structure incentivizes reaching the goal quickly via an efficient path, penalizing meandering or slow progress.
3. **Time Constraint:** A time step threshold is introduced to penalize excessively long exploration or travel times, which are considered mission failures. Furthermore, recent studies have proven that designing multi-component reward structures—which proactively evaluate safe, open spaces and penalize risky proximity—significantly enhances navigation stability in constrained environments [17].

**Figure 3:**
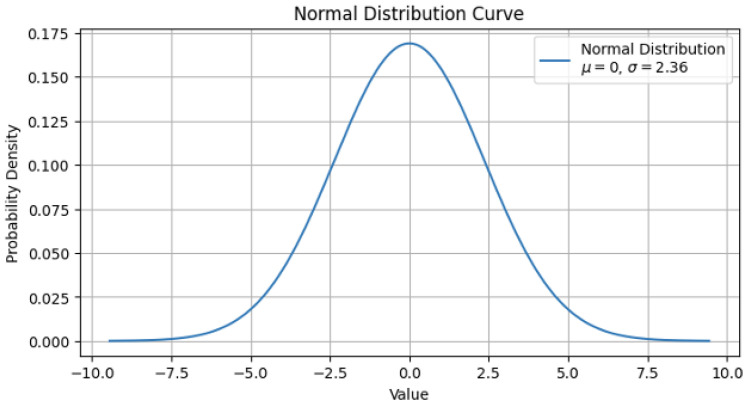
Normal Distribution Graph for Distance Reward. Remaining distance approaches zero (the goal).

To accelerate convergence, we designed a distance-based reward (Figure 3) that increases significantly as the robot approaches the goal, utilizing an exponential function that follows the shape of a normal distribution curve [18]. The Gaussian-shaped reward function plays a crucial role in maintaining learning stability. By providing a smooth gradient of rewards based on a normal distribution, the model avoids sudden, extreme shifts in Q-values during updates, which effectively mitigates the risk of catastrophic bias when coupled with our prioritization mechanism. The general formula for a normal distribution is given in Equation (11).

(11) $$f\left(x\right)=\frac{1}{\sqrt{2\pi \sigma^{2}}}e^{-\frac{{\left(x-\mu \right)}^{2}}{2\sigma^{2}}}$$

In our implementation, the normalization constant $\frac{1}{\sqrt{2\pi \sigma^{2}}}$ is handled as part of a tunable gain hyperparameter. The standard deviation, σ, is set to one-third of the total initial distance, creating a reward curve akin to a normal distribution where the starting distance corresponds to approximately 3σ from the mean (the goal).

To reward path efficiency, we calculate the difference between the total initial Euclidean distance and the remaining distance, while also penalizing inefficient travel over time [19]. Finally, a strong penalty is imposed for selecting paths that lead to dead ends or fail to progress towards the goal.

The equations satisfying these conditions are defined below. The total reward, *R*, is the combination of these components as expressed in Equation (15).

The **distance reward** ($\eta_{d}$) provides an exponentially increasing reward as the robot nears the goal:

(12) $$\eta_{d}=\left\{\begin{array}{cc} k_{d}\cdot \exp\left(-\frac{d_{\mathrm{remain}}^{2}}{2\sigma^{2}}\right), & \mathrm{if}\;\mathrm{no}\;\mathrm{collision}\\ -k_{d}, & \mathrm{if}\;\mathrm{collision}\;\mathrm{occurs} \end{array}\right.$$

The **progress reward** ($\eta_{p}$) encourages forward movement toward the goal while penalizing the time taken:

(13) $$\eta_{p}=k_{p}\cdot \left(\left(d_{\mathrm{total}}-d_{\mathrm{remain}}\right)-\beta \cdot t_{\mathrm{step}}\right)$$

The **time penalty** ($\eta_{t}$) penalizes the agent for exceeding a predefined time step limit:

(14) $$\eta_{t}=\left\{\begin{array}{cc} t_{\mathrm{step}}-t_{\mathrm{threshold}} & \mathrm{if}\;t_{\mathrm{step}}>t_{\mathrm{threshold}}\\ 0 & \mathrm{else} \end{array}\right.$$

The **final reward**, *R*, is the sum of these components:

(15) $$R=\eta_{d}+\eta_{p}-\eta_{t}$$

Here, $\beta =\frac{d_{\mathrm{total}}}{t_{\mathrm{threshold}}}$. This setting ensures that if the time step limit is exceeded, the negative reward for the elapsed time will outweigh the positive reward for progress made. The terms $k_{d}$ and $k_{p}$ are hyperparameters tuned experimentally to balance the influence of each reward component.

**Figure 4:**
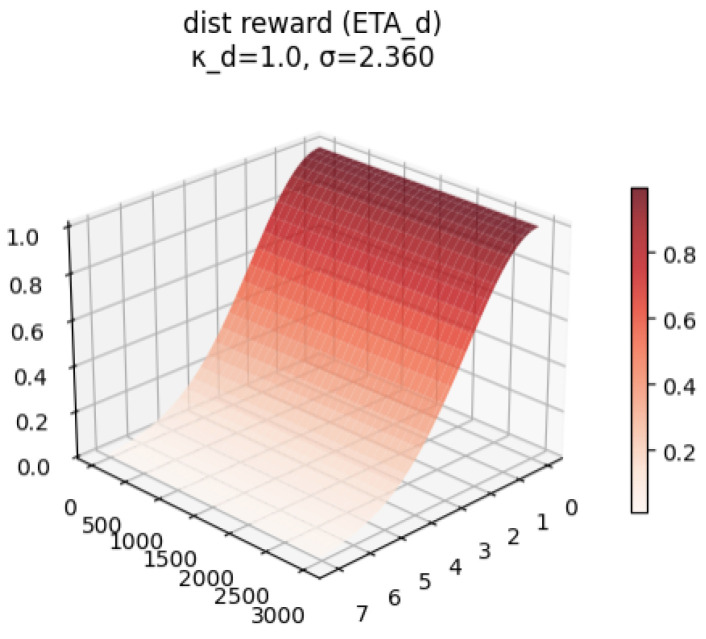
Distance reward ($\eta_{d}$) as a function of remaining distance.

**Figure 5:**
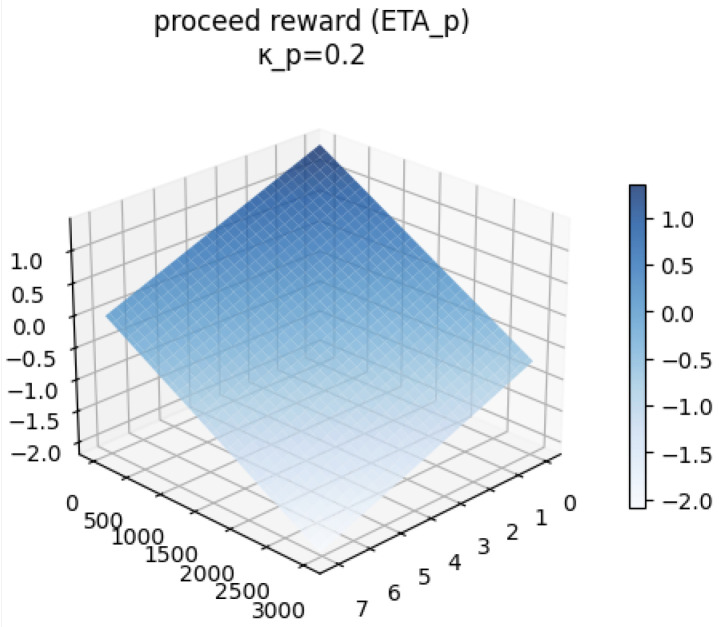
Progress reward ($\eta_{p}$) as a function of time steps.

**Figure 6:**
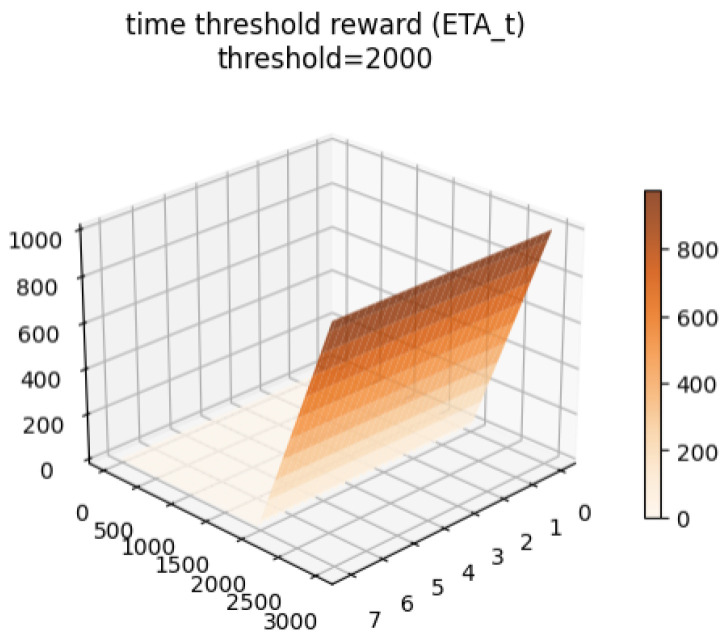
Time penalty ($\eta_{t}$) applied after exceeding the step threshold.

**Figure 7:**
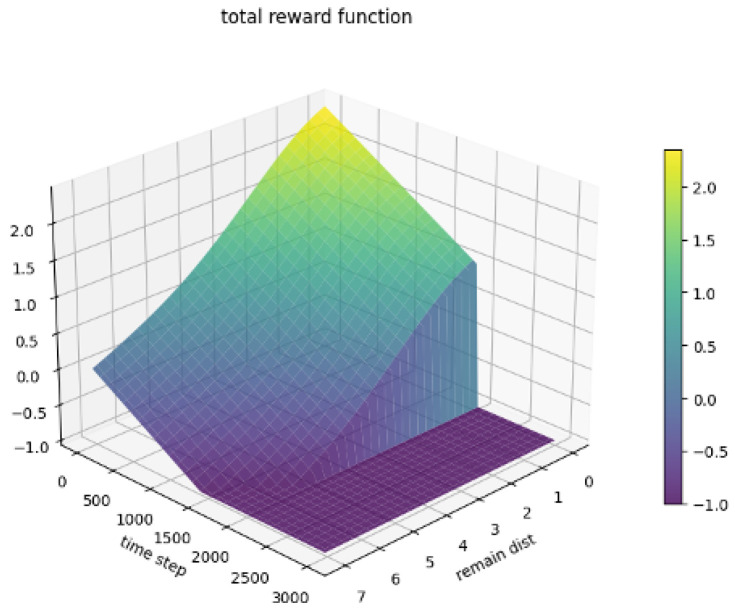
Total proposed reward (*R*) combining all components.

The behavior of each reward component and the total reward are visualized conceptually in Figure 4, Figure 5, Figure 6 and Figure 7.

Through extensive simulation, we set the optimal hyperparameter values as follows:

- **Distance Gain ($k_{d}$):** Determines the magnitude of the attractive signal towards the goal. Fixed at $k_{d}=1.0$.
- **Standard Deviation (σ):** Controls the spread of the Gaussian distribution. Set dynamically to 1/3 of the initial Euclidean distance ($D_{init}/3$) for each episode.
- **Progress Gain ($k_{p}$):** Regulates the trade-off between navigation speed and safety. Selected as $k_{p}=0.5$.

### 3.3. Reward-Based Prioritized Experience Replay

Reinforcement learning networks learn from experiences—transitions of (state, action, reward, next state)—generated through exploration. Strategically selecting which experiences to train on, rather than sampling them uniformly at random, can significantly increase learning efficiency. Uniform sampling ignores the relative importance of different experiences, which can negatively impact both the speed and performance of learning.

By assigning sampling priorities based on specific criteria, we can focus the training on more informative experiences. Recent approaches have demonstrated that coupling specialized network architectures or shaped reward functions with prioritized experience replay significantly accelerates convergence in complex obstacle environments [20]. Prioritized Experience Replay (PER) [21] assigns higher priority to experiences with a larger TD-error. The probability of sampling the *i*-th experience in standard PER is given by Equation (16):

(16) $$P\left(i\right)=\frac{p_{i}^{\alpha }}{\sum_{k}p_{k}^{\alpha }}$$

Here, the priority $p_{i}$ is typically $|TD-error_{i}|+\epsilon$. However, this non-uniform sampling introduces a bias into the learning updates. To correct for this bias, importance-sampling (IS) weights are used to scale the TD-errors during the network update. The weight for the *i*-th sample is defined in Equation (17):

(17) $$w_{i}={\left(\frac{1}{N}\cdot \frac{1}{P(i)}\right)}^{\beta }$$

(18) $$\delta =\left(r+\gamma Q_{\mathrm{target}}\left(s^{\prime },a^{\prime }\right)\right)-Q_{\mathrm{current}}\left(s,a\right)$$

While standard PER relies on TD-errors to prioritize ‘surprising’ transitions, it exhibits critical limitations during the early stages of training. Because the Q-function is unstably initialized, the TD-error may fail to accurately reflect the true importance of an experience and is often heavily corrupted by noise. In contrast, our proposed reward-based PER leverages the raw reward signal, which serves as an absolute ‘ground truth’ directly from the environment. Particularly in mobile robot navigation tasks, prioritizing transitions with low or negative rewards—which represent critical failures such as collisions—forces the agent to rapidly correct unsafe behaviors. This approach significantly enhances both the safety and the convergence speed of the learning process. To incorporate this raw feedback signal directly, we modify the PER framework to define the priority based on the reward value itself, rather than the TD-error.

The rationale behind this modification diverges slightly from traditional PER. In navigation tasks, high rewards indicate that the agent is already traversing an optimal or near-optimal path. Consequently, these experiences represent successful behaviors that require less frequent revisiting. Conversely, steps yielding low or negative rewards (e.g., collisions, inefficient wandering) represent critical failures or suboptimal behaviors that the policy must urgently correct. By sampling these low-reward transitions more frequently, the agent concentrates its learning capacity on rectifying its mistakes rather than redundantly reinforcing already successful behaviors. Reward-based PER ensures the agent learns more frequently from critical failures, which is essential for safety-critical tasks like robot navigation. The priority probability P(i) is redefined as shown in Equation (19):

(19) $$P\left(i\right)=\frac{(-r_{i}+{\left|\min\left(r\right)|\right)}^{\alpha }}{\sum_{k}\left((-r_{k}+{\left|\min\left(r\right)|\right)}^{\alpha }\right)+\epsilon }$$

In this formulation, the priority is calculated based on the negative reward, $-r_{i}$. The term |min(r)| (the absolute value of the minimum reward in the buffer) is calculated globally over the entire replay buffer rather than per-minibatch to maintain a stable global priority distribution and ensure convergence. A small constant ϵ (strictly set to $1\times 10^{-5}$) is added to the denominator exclusively to prevent zero-division errors when all priorities are zero; A small constant ϵ is added to ensure all transitions have a non-zero probability of being sampled. Furthermore, we continue to employ Importance Sampling (IS) weights to mathematically correct the bias introduced by prioritized sampling, as shown in Equation (17).

The parameter β is initially set to a low value (e.g., 0.4) to prioritize learning speed and is annealed to 1.0 as training progresses to reduce bias and ensure convergence. The entire process is summarized in Algorithm 1.
**Algorithm 1** Reward-based Prioritized Experience Replay  1:**Input:** Replay buffer size *N*, priority exponent α, importance sampling exponent β, batch size *k*  2:Initialize prioritized replay buffer *D* and priority list $P_{list}$  3:**for** episode = 1 to M **do**  4:   Initialize environment and state $s_{t}$  5:   **for** t = 1 to $T_{\mathrm{limit}}$ **do**  6:      Select action $a_{t}$ based on current policy with exploration noise  7:      Execute $a_{t}$, observe next state $s_{t+1}$, reward $r_{t}$, done flag $d_{t}$  8:      Store experience $\left(s_{t},a_{t},r_{t},s_{t+1},d_{t}\right)$ in *D*  9:      Store priority based on transformed reward $-r_{t}$ in $P_{list}$10:      **if** learning step is due **then**11:     Compute minimum reward in buffer: $r_{\min}=\left|\min\left(P_{list}\right)\right|$12:     For each experience *i* in buffer, compute probability: $P\left(i\right)=\frac{{\left(-r_{i}+r_{\min}\right)}^{\alpha }}{\sum_{k}{\left(-r_{k}+r_{\min}\right)}^{\alpha }+\epsilon }$13:     Sample a mini-batch of *k* experiences from *D* according to probabilities P(i)14:     For each sampled experience *i*, compute IS weight: $w_{i}={\left(\frac{1}{N\cdot P(i)}\right)}^{\beta }$15:     Normalize weights: $w_{i}\leftarrow \frac{w_{i}}{\max_{k}w_{k}}$16:     Compute TD-error $\delta_{i}$ for each experience in the mini-batch17:     Update network parameters using weighted loss: $\nabla L=\sum w_{i}\delta_{i}^{2}$18:     Periodically update target networks19:      **end if**20:      $s_{t}\leftarrow s_{t+1}$21:   **end for**22:**end for**

### 3.4. LiDAR Point Cloud Preprocessing

To utilize raw point cloud data from a 3D LiDAR sensor for reinforcement learning, it is essential to convert its unstructured format into a structured, fixed-size representation suitable for a neural network input. We employ a two-stage technique that first regularizes the point cloud into an image-like representation and then downsamples it into a low-dimensional tensor.

#### 3.4.1. Projection to 2D Angular Grid

**Figure 8:**
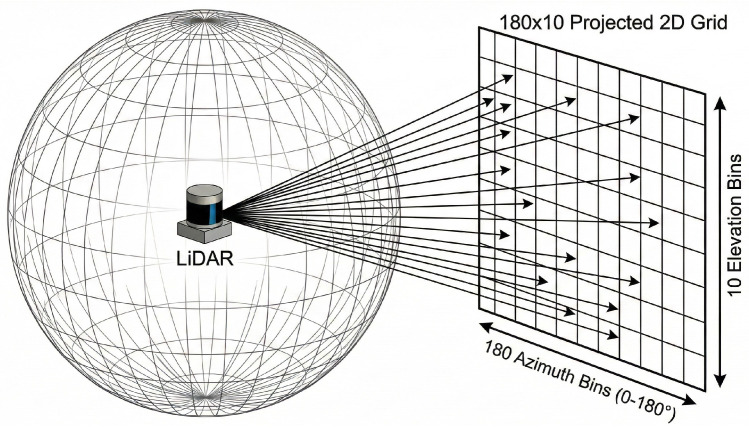
Conceptual partitioning of the LiDAR’s field of view into an angular grid.

The first stage involves projecting the 3D points onto a 2D grid. The horizontal axis corresponds to the azimuth angle, and the vertical axis corresponds to the elevation angle. This process involves partitioning the sensor’s horizontal field of view (72 bins) and its vertical field of view (10 bins) into a grid of discrete angular cells, as conceptually illustrated in Figure 8.

**Figure 9:**
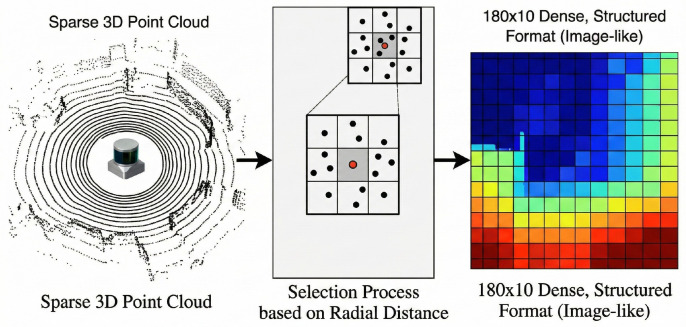
Voxelization process from raw point cloud to an image-like state representation.

For each cell, we identify all LiDAR points that fall into it and select representative points based on radial distance. This effectively transforms the sparse 3D point cloud into a dense, structured format. The preprocessing pipeline is summarized in Figure 9.

#### 3.4.2. Angular-Based Point Selection for State Representation

To facilitate effective reinforcement learning, it was imperative to drastically reduce the number of LiDAR points while preserving their semantic meaning. We downsample the points by selecting a fixed number of critical points from angularly equidistant sectors in the robot’s field of view.

**Figure 10:**
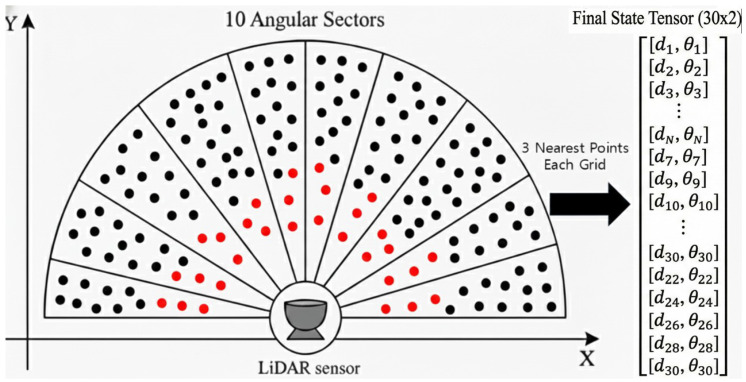
Partitioning the flattened point cloud into 10 angular sectors. Conceptually, the nearest points are selected to form the final state representation.

The horizontal field of view is partitioned into 10 sectors of equal angular width. For each sector, we specifically select the 3 strictly nearest points to the robot. Prioritizing the nearest distances ensures that detailed, critical obstacle information directly within the robot’s path is never occluded or discarded during downsampling. While Figure 10 conceptually illustrates the selection of a single nearest point for visual clarity, our robust implementation relies on extracting the 3 nearest points.

This method results in a total of 30 selected points (10 sectors × 3 points), retaining 2 features for each selected point (e.g., distance and relative angle) to form a state component of shape 30×2. This tensor is then combined with the robot’s navigation state to form the final input to the RL agent.

### 3.5. Hybrid Control: Integrating Path Planning with Reinforcement Learning

Reinforcement learning networks determine actions stochastically based on the actor’s policy, making them highly sensitive to initial parameters. Without an effective value baseline, an initial policy with high variance can cause overly aggressive updates, leading to learning failure.

In this research, we propose an alternative approach: supplement the weaknesses of RL by using a traditional path planner to guide the learning process in real time. Instead of letting the planner’s actions dictate exploration directly—which risks trapping the agent in a local optimum—we indirectly influence learning by modifying the TD-target. The path planning algorithm generates a “guiding” action, which is used to calculate an alternative target Q-value.

The standard TD-target in TD3 is calculated as follows:

(20) $$\begin{array}{cc} Q_{\mathrm{target}} & =\min(Q_{\theta_{1}^{\prime }}\left(s^{\prime },a^{\prime }\right),Q_{\theta_{2}^{\prime }}\left(s^{\prime },a^{\prime }\right))\\ \mathrm{where}\;a^{\prime } & =\pi_{\phi^{\prime }}\left(s^{\prime }\right) \end{array}$$

**Figure 11:**
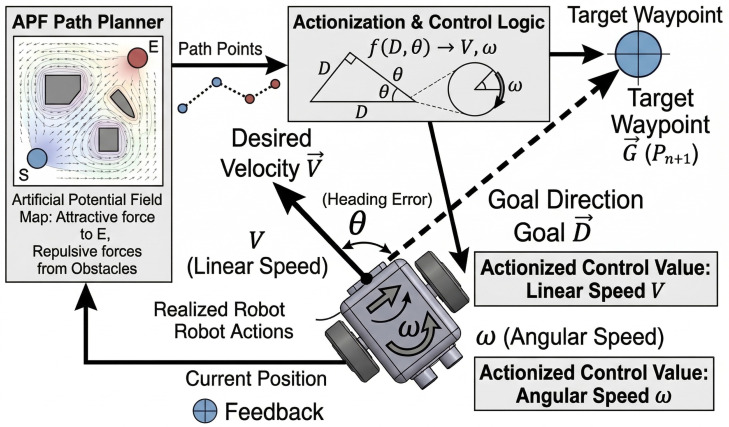
Converting a planner-generated waypoint into a continuous angular velocity action for the RL agent.

We selected APF as our classical path planner. As illustrated in Figure 11, we calculate the direction vector from the robot’s current position to the waypoint, and the normalized angle serves as the action, ω.

This planner-generated action is used to calculate a candidate target Q-value. The final target Q-value is determined by taking the maximum between the value from the original TD3 policy and the value from the path planner’s proposed action, as shown in Equation (21).

(21) $$\begin{array}{cc} Q_{\mathrm{target}}=\max\{ & \min\left(Q_{\theta_{1}^{\prime }}\left(s^{\prime },\pi_{\phi^{\prime }}\left(s^{\prime }\right)\right),Q_{\theta_{2}^{\prime }}\left(s^{\prime },\pi_{\phi^{\prime }}\left(s^{\prime }\right)\right)\right),\\ \; & \min\left(Q_{\theta_{1}^{\prime }}\left(s^{\prime },a_{\mathrm{planner}}\right),Q_{\theta_{2}^{\prime }}\left(s^{\prime },a_{\mathrm{planner}}\right)\right)\} \end{array}$$

It is crucial to address the theoretical implications of introducing a max operator into the target calculation, as it might ostensibly seem to contradict the overestimation suppression philosophy of TD3. In standard Q-learning, the Bellman optimality target introduces a severe positive bias due to the maximization over the entire continuous action space: $y=r+\gamma \max_{a^{\prime }}Q\left(s^{\prime },a^{\prime }\right)$. TD3 mitigates this by applying a min operator over two independently initialized critic networks for a single policy action. In our proposed framework, we strictly decouple the *value estimation bias control* from the *action candidate selection*.

Specifically, we define a discrete candidate set containing exactly two actions: $A_{c}=\left\{\pi_{\phi^{\prime }}\left(s^{\prime }\right),a_{\mathrm{planner}}\right\}$. Before any maximization occurs, the value of each candidate action is independently and conservatively evaluated using the standard Clipped Double-Q mechanism ($\min(Q_{1},Q_{2})$). Consequently, the modified Bellman target can be mathematically expressed as:

(22) $$y=r+\gamma \max_{a\in A_{c}}\left(\min_{j=1,2}Q_{\theta_{j}^{\prime }}\left(s^{\prime },a\right)\right)$$

Because the max operator is strictly confined to selecting between two conservatively bounded estimates, rather than searching an unbounded Q-value landscape, it functions solely as a behavioral selection mechanism. While this represents a practical compromise that marginally relaxes TD3’s strict pessimism to leverage classical heuristic guidance, it mathematically preserves the foundational overestimation suppression architecture.

**Figure 12:**
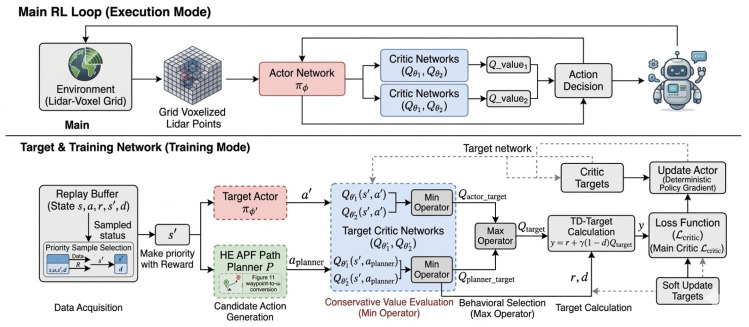
The proposed hybrid architecture combining path planning and reinforcement learning. The planner’s action is used to generate a candidate Q-target, guiding the update of the critic and actor networks.

By setting the target Q-value in this manner, the agent is guided to follow the more promising action. A diagram illustrating this hybrid control architecture is shown in Figure 12, and the complete process is detailed in Algorithm 2.
**Algorithm 2** TD3 with Path Planning-enhanced Target Q  1:Initialize actor $\pi_{\phi }$, critics $Q_{\theta_{1}},Q_{\theta_{2}}$ and their target networks $\pi_{\phi^{\prime }},Q_{\theta_{1}^{\prime }},Q_{\theta_{2}^{\prime }}$  2:Initialize replay buffer *D* and path planner *P*  3:**for** each training iteration **do**  4:   Sample a mini-batch of transitions $\left(s,a,r,s^{\prime },d\right)$ from *D*  5:   Compute the planner’s action for the next state: $a_{\mathrm{planner}}\leftarrow P\left(s^{\prime }\right)$  6:   Compute the actor’s target action for the next state: $a^{\prime }\leftarrow \pi_{\phi^{\prime }}\left(s^{\prime }\right)+\mathrm{noise}$  7:   Compute the Q-target from the planner’s action:     $Q_{\mathrm{planner}\_\mathrm{target}}=\min\left(Q_{\theta_{1}^{\prime }}\left(s^{\prime },a_{\mathrm{planner}}\right),Q_{\theta_{2}^{\prime }}\left(s^{\prime },a_{\mathrm{planner}}\right)\right)$  8:   Compute the Q-target from the actor’s action:     $Q_{\mathrm{actor}\_\mathrm{target}}=\min\left(Q_{\theta_{1}^{\prime }}\left(s^{\prime },a^{\prime }\right),Q_{\theta_{2}^{\prime }}\left(s^{\prime },a^{\prime }\right)\right)$  9:   Select the superior target:     $Q_{\mathrm{target}}=\max\left(Q_{\mathrm{planner}\_\mathrm{target}},Q_{\mathrm{actor}\_\mathrm{target}}\right)$10:   Compute the final TD-target: $y=r+\gamma \left(1-d\right)Q_{\mathrm{target}}$11:   Update critics by minimizing the loss: $L_{\mathrm{critic}}=\sum {\left(Q_{\theta_{j}}\left(s,a\right)-y\right)}^{2}$12:   **if** it is time to update the actor **then**13:   Update actor policy using the deterministic policy gradient14:   Soft update all target networks15:   **end if**16:**end for**

## 4. Experiments

### 4.1. Experimental Setup and Environments

The experiments in this study were conducted in NVIDIA’s Isaac Sim, a high-fidelity robotics simulation platform that offers realistic physics and rendering. To assess how effectively the mobile robot executed its intended movements, we recorded the average and maximum cumulative reward values over evaluation episodes. We also tracked the average and maximum Q-values, as well as the average critic network loss.

**Figure 13:**
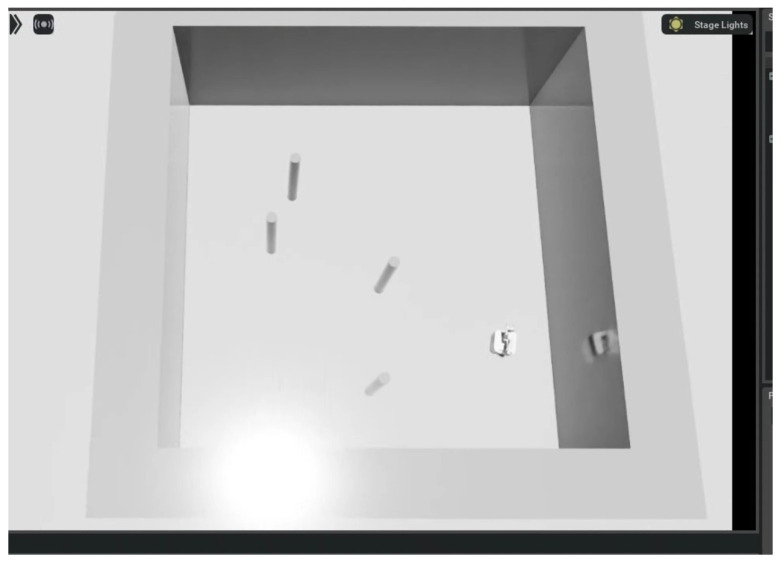
The mobile manipulator platform used in the Isaac Sim simulations.

All simulations were performed on a desktop computer equipped with an NVIDIA RTX 3060 GPU. The robot platform utilized was a mobile manipulator based on the AgileX (AgileX Robotics, Shenzhen, China) Tracer Mini, as depicted in Figure 13.

To ensure statistical significance and address the sensitivity of reinforcement learning to random initialization and exploration noise, every training result and curve reported in this study is obtained by averaging over 10 independent training runs with different random seeds. The TD3 agent was trained with a learning rate of 0.0003, a discount factor (γ) of 0.9, a soft target update rate (τ) of 0.005, and a mini-batch size of 512. To maintain stable exploration and target smoothing, the policy noise was set to 0.2 (clipped at 0.5), and the policy network was updated every 2 critic updates. For the traditional APF planner, the attractive gain ($k_{att}$), repulsive gain ($k_{rep}$), and repulsive influence distance ($d_{0}$) were empirically set to 1.0, 0.8, and 1.0 m, respectively.

### 4.2. Results in Isaac Sim Environment

The training environment consisted of a moderately complex indoor space with static obstacles. To ensure a strictly fair comparison, we applied the exact same action mapping scheme (controlling only the angular velocity, with linear velocity heuristically determined) and identical state dimensions to the baseline TD3 model.

**Figure 14:**
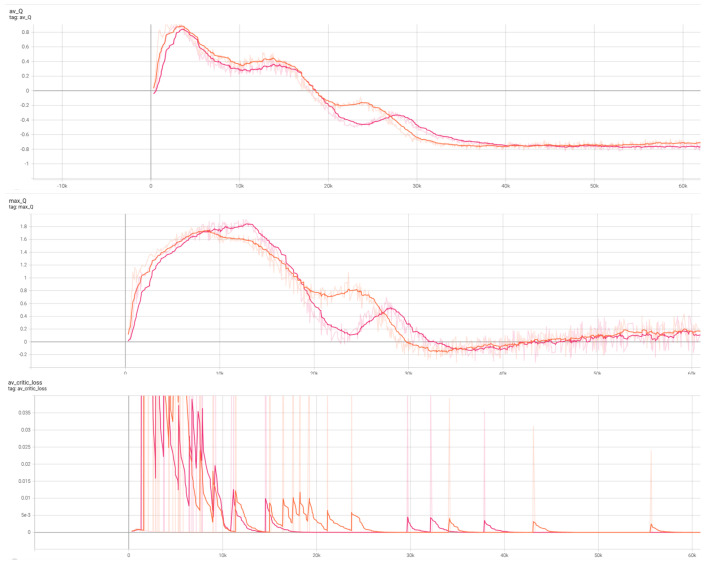
Training results comparing baseline TD3 (red) and TD3 + APF (orange) without PER. Solid lines represent the mean of 10 runs, while shaded areas denote the standard deviation (σ or 95% confidence interval). The plots show (**top**) Average Q-value, (**middle**) Maximum Q-value, and (**bottom**) Critic Loss.

We first conducted a priority comparison of performance based on the integration of the Artificial Potential Field (APF). As shown in Figure 14, the red curve represents the conventional TD3 algorithm, and the orange curve represents TD3 with APF applied. It is evident that TD3 + APF achieves slightly better results.

**Figure 15:**
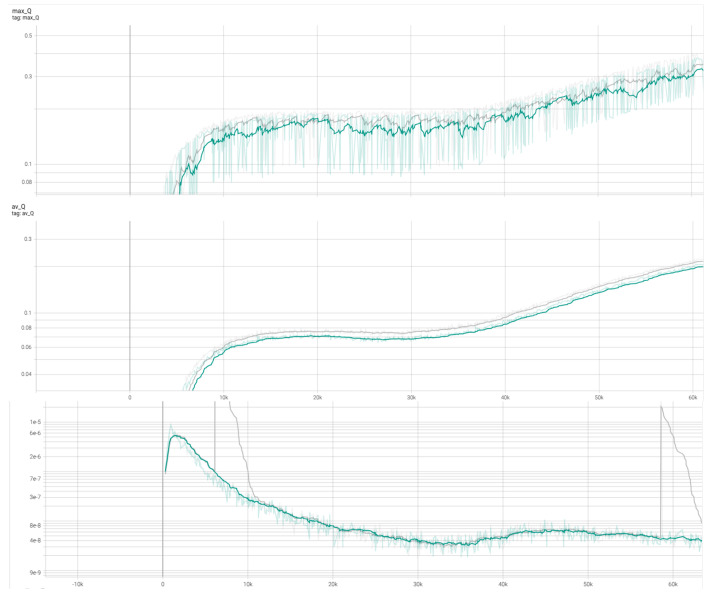
Training results comparing TD3 + PER (green) and TD3 + PER + APF (gray). Solid lines represent the mean of 10 runs, while shaded areas denote the standard deviation. The plots show (**top**) Maximum Q-value, (**middle**) Average Q-value, and (**bottom**) Critic Loss.

Figure 15 illustrates the results with PER applied. The gray curve represents the model with APF, while the green curve represents the model without APF. The overall results demonstrate superior evaluation metrics compared to Figure 14, confirming that our reward-based PER introduces a substantial improvement in learning performance.

To empirically validate our theoretical assertion that the hybrid Q-target calculation does not reintroduce catastrophic overestimation bias, we analyzed the Q-value trajectories and critic loss stability during training. As depicted in Figure 14 and Figure 15, the inclusion of the APF-guided max operator (orange and gray curves) does not lead to Q-value divergence or runaway positive bias. Instead, the maximum and average Q-values converge smoothly and stabilize earlier than the baseline. Furthermore, the critic loss remains stable and avoids extreme early-training variance, confirming that our approach safely accelerates learning through heuristic guidance without compromising the operational stability of the critic network.

Table 1 presents a quantitative comparison of the core evaluation metrics across the tested architectures. Rather than utilizing a highly variable success rate metric, we strictly evaluated the networks based on their Average Reward, Maximum Reward, and Converged Average Q-value. The Average Q-value is a particularly strong indicator of stability, reflecting the agent’s expected return across diverse and randomized environmental states.

The standard TD3 Baseline exhibits an average reward of −35.22, suggesting that it frequently gets trapped in local minima or terminates early due to collisions. In stark contrast, the Proposed Method achieves a significantly higher average reward of 705.1 and a peak reward of 1078. Furthermore, the higher converged Q-value of the proposed architecture demonstrates a more reliable and robust value estimation. This confirms that the proposed hybrid framework, driven by reward-based PER and APF guidance, effectively navigates to the target consistently without succumbing to the instability often observed in standard RL.

## 5. Conclusions

This paper aimed to design a robust and efficient mobile robot navigation system based on a deep reinforcement learning framework. The primary objective was to enable a robot to safely and reliably navigate to a target location in an environment with obstacles, serving as a foundational capability for more complex mobile manipulation tasks.

To this end, we sought to improve the overall learning efficiency and policy robustness of the standard TD3 algorithm by addressing its inherent limitations. Through experiments conducted in the high-fidelity Isaac Sim environment, we demonstrated several key contributions. First, we enhanced performance by integrating traditional APF path planning to mitigate the performance degradation caused by stochastically generated actions early in training. Second, we boosted learning efficiency by assigning priority to informative experiences based on their negative rewards, concentrating the network’s capacity on rectifying failures while preventing bias through importance-sampling weights. Finally, we developed an effective method to downsample high-dimensional LiDAR point cloud data into a low-dimensional state representation suitable for reinforcement learning.

The significance of this improved TD3-based robot navigation system lies in its ability to perform the entire sequence of perception, judgment, and control within a single, unified network, while leveraging classical methods to bootstrap and stabilize the learning process.

While research on combining path planning and reinforcement learning has existed previously, our approach differs fundamentally in its control hierarchy. Prior studies have typically employed a path planner as the primary controller, with RL acting as a supervisor. In contrast, our algorithm reverses this: the RL Actor network directly controls the robot, and path planning is integrated as a ‘teacher’ to improve the learning of the value function. We have demonstrated that this reversal of control priority can effectively overcome the respective shortcomings of each method—using RL’s exploration to escape the planner’s local minima, and using the planner’s stability to guide RL’s initial random exploration.

Looking forward, this research can be extended in several promising directions. The robustness of the network was established in a relatively simple, static environment. Future work will involve applying the methodology developed in this study within a curriculum learning framework to enable generalization to more complex and dynamic environments. Furthermore, deploying and validating the trained policy on a real-world robotic platform will be a crucial next step to bridge the sim-to-real gap and demonstrate the practical utility of our proposed hybrid approach.

## Figures and Tables

**Table 1:** Quantitative comparison of evaluation metrics across evaluated algorithms in the Isaac Sim environment. Results are reported as Mean ± Standard Deviation over 10 random seeds.

Algorithm	Avg Reward	Max Reward	Avg Q-Value
TD3 Baseline (Grid State)	−35.22 ± 48.5	973.6	420.5 ± 31.2
**Proposed Method**	** 705.1±42.3 **	** 1078.0 **	** 815.3±15.6 **

## Data Availability

The original contributions presented in this study are included in the article. Further inquiries can be directed to the corresponding author.
